# A man, a gadget, a legacy

**DOI:** 10.1590/2176-9451.20.2.010-011.edt

**Published:** 2015

His work is not the price he pays for being a man, but a way of loving and helping the world to become a better place. Thiago de Mello, Amazonian poet.

The year was 1995. At the San Francisco airport hall, we awaited the flight to Los Angeles, USA, where we would attend another American congress. At halfway point, a group of orthodontists was talking to a gray-haired gentleman. Sited, he demonstrated how to build an appliance he had created - a Lego-like appliance. His smooth and rather weak voice was indeed persevering. The gray-haired gentleman was Carlos Martins Coelho Filho. I have to confess that, the first time we met, his human figure and smooth voice commanded my admiration more than the appliance, the MPA III,^1^ did. 

A few years later, the MPA IV entered the orthodontic field^2^ at the same time I established a closer relationship with Prof. Carlos Coelho. A delightful human being who emanated a dazzling light, infinitely amplified by the fact that it originated from a modest, humble and unquestionably intelligent man. As time went by, we became friends. 

Born in São Luís, Maranhão (Brazil), Carlos received his Master's degree in Orthodontics from the Piracicaba Dental School (UNICAMP). Before that, he used to be an electronics technician. He also increased his income by giving English lessons. He once told me that, as soon as he was approved by the Masters course, he called professor Muller de Araújo, former head of the program, feeling sorry for having difficulties to attend it on the date set for the beginning of the course. The hurdle was financial. He needed time to sell his small electronics workshop and his old Beetle. He never expected to be approved, given the fierce competition and his own personal difficulties. To his great surprise, Prof. Araújo not only accepted his postponed arrival, but also highlighted his qualities and told him it was his own wish, Prof. Araújo's, to count with the presence of that student, given the sacrifice he would make to take the course. Carlos told me this story a few times and I never interrupted him, as his eyes sparkled with the memories of such a dear professor. Perfume of gratitude.

He also told me about his first contact with a dental spot welder. It was an imported equipment that belonged to one of his professors. At that time, the device was rather uncommon in Brazil, but the dream of any orthodontist. Carlos applied his knowledge about electronics to create a copy of the welding machine based on an analysis of the imported equipment. The orders boosted dramatically. 

After receiving his Master's degree, Carlos returned to Maranhão and launched his career as a professor at the School of Dentistry at Universidade Federal do Maranhão. He got married to Rosa with whom he had two children: Eduardo and Fábio. However, it was in private practice that Prof. Coelho initiated the conception of his masterpiece, a third child, so to speak: the mandibular protraction appliance (MPA). A gadget, as he would say, to promote mandibular advancement and treat Class II malocclusion. What most impressed me was the fact that Carlos graduated at a time when moving the mandible forward sounded as heresy in the field of Orthodontics. Only a few orthodontists ventured on trial and those who did were condemned. He was, thus, an atemporal professional far from any kind of prejudice. 

When he first began developing his masterpiece, Carlos realized how fragile the system was and reported numerous cases of breakage. Nevertheless, he humbly accepted its limitations and, with perseverance and cleverness, improved his work and gradually developed MPA model IV, more stable and comfortable. 

The MPA, or some of its adaptations, has been currently used by a great number of Brazilian orthodontists. However, despite several international publications, the appliance has not become well-known outside Brazil.^1^^-^ 7 It is not hard to understand the reasons why. One day, I asked him whether he ever thought about commercializing his invention through a large company. Carlos, in his infinite humbleness, as much as to cause us anger, reported that an American company had made him a commercial offer which he refused. He would like his masterpiece to be freely accessed by any colleague: a "do it yourself". Carlos' inventor brain was not blessed with a business cortex. 

Brazilian Orthodontics owes a great deal of gratitude to the inventor Coelho Filho; while we, as his admirers, owe an immense debt to this *nordestino*: an auspicious clinician and an altruistic human being. A professional of untouched conduct and a friend of inspiring sagacity and generosity, whose legacy exceeds these pages or all volumes of this journal... But certainly fit his heart. He simply presented us, as a divine gift, with what he created.

Nobility is engraved in the step-by-step and little-by-little of his work; short stories that render a man great and compose such an indestructible legacy.

David Normando - editor-in-chief (davidnormando@hotmail.com)
