# Supplementary material for: A man, a gadget, a legacy
Source: Dental Press J Orthod. 2015 Mar-Apr;20(2):10–1. doi: 10.1590/2176-9451.20.2.010-011.edt (PMC4445218; doi:10.1590/2176-9451.20.2.010-011.edt)
Supplement: Translation

# Translation: Um homem, uma engenhoca, um legado

O seu trabalho não é a pena que paga por ser homem, mas um modo de amar e de ajudar o mundo a ser melhor. Thiago de Mello, poeta amazonense.

O ano era 1995. Aguardávamos, no saguão do aeroporto de San Francisco, o voo para Los Angeles, EUA, porventura para mais um congresso americano. A meia-distância, um grupo de ortodontistas conversava com um senhor grisalho. Sentado, ele demonstrava como construir um aparelho que havia criado - uma espécie de Lego. A voz suave, fraca até, era cheia de perseverança. O tal grisalho era Carlos Martins Coelho Filho. Naquele primeiro contato, confesso que a figura humana e a suavidade da voz causaram-me mais admiração do que o próprio aparelho, o APM III^1^. 

Anos mais tarde, o modelo APM IV entrava na seara ortodôntica^2^, ao mesmo tempo em que me aproximei do Prof. Carlos Coelho. Ser encantador, que emanava luz de brilho intenso, infinitamente amplificada pelo fato de partir de um homem simples, humilde e de inteligência inconteste. Com o tempo, tornamo-nos amigos. 

Maranhense de São Luís, Carlos fez mestrado em Ortodontia na Faculdade de Odontologia de Piracicaba. Antes, ele era técnico em eletrônica. Também complementava a renda dando aulas de inglês. Contou-me que, ao ser aprovado no seu curso de mestrado, ligou para o professor Muller de Araújo, então coordenador do programa, lamentando que, a despeito da aprovação, teria dificuldades para estar presente na data definida para início do curso. O empecilho era financeiro, pois precisaria de tempo para vender a pequena oficina de eletrônica e o seu fusquinha. Não esperava ser aprovado, dada a grande concorrência e suas dificuldades. Em grata surpresa, o Prof. Araújo, além de aceitar a sua prorrogação, enalteceu as suas qualidades e disse-lhe que era ele, o Prof. Araújo, quem fazia questão da presença daquele aluno, dado o sacrifício que faria para seguir o curso. Carlos contou-me essa história algumas vezes, e eu nunca o interrompi porque os seus olhos sempre brilhavam com a lembrança do estimado professor, um perfume de gratidão.

Contou-me também sobre o seu primeiro contato com uma máquina de solda a ponto. Era um equipamento importado, pertencente a uma de suas professoras. Na época, o dispositivo era pouco comum no Brasil e sonho de consumo de qualquer ortodontista. Carlos usou os seus conhecimentos de eletrônica e fez, ele mesmo, uma cópia da máquina de solda, a partir da análise do equipamento importado. Descarrilaram-se encomendas. 

Após a conclusão do mestrado, Carlos retornou ao Maranhão e ingressou como professor na Faculdade de Odontologia da Universidade Federal do Maranhão. Casou-se com Rosa, com quem teve dois filhos, Eduardo e Fábio. Mas foi na prática privada que o Prof. Coelho iniciou a concepção da sua maior obra, um terceiro filho, podemos assim dizer: o aparelho de protração mandibular (APM). Uma engenhoca, como ele mesmo dizia, que promovia o avanço da mandíbula no intuito de tratar as más oclusões de Classe II. O que me impressionou foi o fato de Carlos ter se formado em uma época na qual avanços mandibulares soavam como heresias dentro da Ortodontia. Os poucos ortodontistas que se lançavam à experimentação eram quase execrados do meio. Percebe-se, destarte, um profissional atemporal e distante dos preconceitos.

No início do desenvolvimento da sua obra, Carlos logo percebeu a fragilidade do sistema, relatando constantes quebras. Aceitou com humildade as limitações e, com perseverança e inteligência, melhorou a obra até chegar ao modelo IV, mais estável e confortável. 

Atualmente, o APM, ou algumas de suas modificações, é utilizado por grande parte dos ortodontistas brasileiros. O aparelho não ficou tão conhecido fora do Brasil, a despeito das diversas publicações internacionais^1^^-^ 7. Não é difícil compreender o motivo. Certo dia, perguntei-lhe se nunca havia pensado em comercializar a sua invenção por meio de uma grande empresa. Carlos, na sua infinita humildade - de dar raiva até -, relatou que uma grande companhia americana já havia lhe oferecido uma proposta comercial, a qual recusara. Ele gostaria que a sua obra fosse de livre acesso a qualquer colega, no modelo "faça você mesmo". Ao cérebro inventor de Carlos não foi concedido o córtex empresarial. 

A Ortodontia brasileira deve muito ao inventor Coelho Filho; e nós, seus admiradores, temos uma dívida imensurável com esse nordestino, clínico alvissareiro e ser humano altruísta. Um profissional de conduta ilibada e um amigo de sagacidade iluminada e fidalguia, cujo legado não cabe nestas páginas ou em todos os volumes deste periódico... Mas no seu coração, esse sim, coube. E ele, simplesmente, nos doou, divinamente, o que criou.

A nobreza estampa-se no passo a passo, no pouco a pouco, e são pequenas histórias que tornam um homem grande e comporão um legado indestrutível.

David Normando - editor-chefe (davidnormando@hotmail.com)
